# Proposal for a New Pathologic Prognostic Index After Neoadjuvant Chemotherapy in Pancreatic Ductal Adenocarcinoma (PINC)

**DOI:** 10.1245/s10434-022-11413-7

**Published:** 2022-03-01

**Authors:** M. Redegalli, M. Schiavo Lena, M. G. Cangi, C. E. Smart, M. Mori, C. Fiorino, P. G. Arcidiacono, G. Balzano, M. Falconi, M. Reni, C. Doglioni

**Affiliations:** 1 Pathology Unit, IRCCS San Raffaele Scientific Institute, Vita-Salute San Raffaele University, Milan, Italy; 2 Department of Medical Oncology, IRCCS San Raffaele Scientific Institute, Pancreas Translational and Clinical Research Centre, Milan, Italy; 3 Medical Physics, San Raffaele Scientific Institute, Milan, Italy; 4 Pancreato-Biliary Endoscopy and Endosonography Division, Pancreas Translational and Clinical Research Centre, San Raffaele Scientific Institute, Vita Salute San Raffaele University, Milan, Italy; 5 Pancreatic Surgery Unit, Pancreas Translational and Clinical Research Centre, IRCCS San Raffaele Scientific Institute, Vita-Salute San Raffaele University, Milan, Italy

## Abstract

**Background:**

Limited information is available on the relevant prognostic variables after surgery for patients with pancreatic ductal adenocarcinoma (PDAC) subjected to neoadjuvant chemotherapy (NACT). NACT is known to induce a spectrum of histological changes in PDAC. Different grading regression systems are currently available; unfortunately, they lack precision and accuracy. We aimed to identify a new quantitative prognostic index based on tumor morphology.

**Patients and Methods:**

The study population was composed of 69 patients with resectable or borderline resectable PDAC treated with preoperative NACT (neoadjuvant group) and 36 patients submitted to upfront surgery (upfront-surgery group). A comprehensive histological assessment on hematoxylin and eosin (H&E) stained sections evaluated 20 morphological parameters. The association between patient survival and morphological variables was evaluated to generate a prognostic index.

**Results:**

The distribution of morphological parameters evaluated was significantly different between upfront-surgery and neoadjuvant groups, demonstrating the effect of NACT on tumor morphology. On multivariate analysis for patients that received NACT, the predictors of shorter overall survival (OS) and disease-free survival (DFS) were perineural invasion and lymph node ratio. Conversely, high stroma to neoplasia ratio predicted longer OS and DFS. These variables were combined to generate a semiquantitative prognostic index based on both OS and DFS, which significantly distinguished patients with poor outcomes from those with a good outcome. Bootstrap analysis confirmed the reproducibility of the model.

**Conclusions:**

The pathologic prognostic index proposed is mostly quantitative in nature, easy to use, and may represent a reliable tumor regression grading system to predict patient outcomes after NACT followed by surgery for PDAC.

**Supplementary Information:**

The online version contains supplementary material available at 10.1245/s10434-022-11413-7.

Pancreatic ductal adenocarcinoma (PDAC) is one of the most lethal malignancies.^1^ Over 50% of patients are metastatic at diagnosis, while only 10–20% of patients are diagnosed with resectable disease. Even though surgery is still the mainstay of therapy, early systemic relapses occur in up to 80% of cases after surgical management intended to be curative.^2^ The rapid appearance of recurrence strongly suggests the presence of subclinical diffusion in early phase disease. Furthermore, due to postoperative complications, a significant percentage of patients are unable to start any kind of chemotherapy that implies significant toxic effects. In the APACT study,^3^ comparing the association of gemcitabine and nab-paclitaxel to gemcitabine alone as adjuvant therapy in resected PDAC patients, only 866 (71%) patients were randomized out of 1226 screened patients. The screening failure was mainly related to evidence of either potential residual or metastatic disease. Moreover, only 69% of randomized patients completed the planned six cycles of chemotherapy (nab-paclitaxel/gemcitabine, 66%; gemcitabine, 71%).^3^

Accordingly, a preoperative therapeutic strategy is worthy of investigation, and several ongoing trials are exploring the role of NACT in early stage PDAC. Neoadjuvant chemotherapy (NACT) has been tested in a few randomized studies, which suggest benefits compared with up-front resection followed by adjuvant therapy.^4,5^ NACT is now considered an acceptable option for treatment of resectable and borderline resectable PDAC by the National Comprehensive Cancer Network (NCCN) guidelines.^6^ However, no information is available on the optimal therapeutic management after surgery for patients who have undergone NACT. This information gap is also due to a lack of prognostic stratification factors in these patients.

NACT is known to induce a spectrum of histological changes in PDAC.^7,8^ Since the 1980s, several groups have proposed histopathological systems for the grading of response to chemo- and radiotherapy, aimed at developing a prognostic tool to guide postsurgical patient management.^9–12^ To date, these schemes have been based on the assessment of the amount of residual tumor, viability, tumor destruction, presence of mucin, and fibrosis. However, there is no international consensus as to which tumor response grading (TRG) system represents the best option.^13^ Indeed, there are many factors that make standardization difficult. First, the method and extent of tissue sampling is rarely specified and probably varies among studies.^13^ In addition, interobserver studies demonstrate poor concordance and a lack of precision and accuracy, calling into question the clinical utility of TRG systems.^7^

Therefore, further studies are needed to develop a reproducible and clinically relevant grading system based on prognostic markers validated for the neoadjuvant setting. The main objective of the present study is to identify objective parameters that predict prognostic value in PDAC patients resected after NACT and can be eventually incorporated into a prognostic index.

## Patients and Methods

### Study Population

The study population consisted of 69 patients with nonmetastatic, resectable or borderline resectable^14^ cytologically confirmed PDAC who received primary combination NACT followed by surgical resection at our Institution between July 2005 and February 2016. To ensure homogeneity of eligibility criteria, treatment, staging and follow-up procedures, data collection and cleaning, only patients who were treated in the context of prospective clinical trials approved by the San Raffaele Scientific Institute Ethics Committee^4,15–17^ (Supplementary Table S1) were considered. We also included 36 patients who were underwent upfront surgery in order to comprehensively compare their histopathological features with patients treated with NACT. Of note, the upfront-surgery group included patients who were randomized in a neoadjuvant versus adjuvant trial^4^ and therefore represent an ideal comparator population because they were not selected on surgical outcome or postoperative recovery. Patients who received NACT were treated with anthracycline-containing (*n* = 50) or taxane-containing (*n* = 19) regimens for 3–6 months, according to the study design. On average, patients who received NACT underwent surgery 52.3 days (range 18–413 days) after the last chemotherapy administration.

### Morphological Evaluation

All surgical specimens were examined and processed according to internal protocols of the pathology unit. The pancreaticoduodenectomy specimens were sampled with the bivalving approach through the common bile duct or by axial sections with en bloc inclusion of peripancreatic tissue. The distal pancreatectomy specimens were sampled by sectioning from the resection margin to the spleen. Total pancreatectomy specimens were sectioned at the level of the isthmus, and then the two halves were sampled as pancreaticoduodenectomy and distal pancreatectomy specimens, respectively. The “tumor bed” identified at the macroscopic level was included entirely and, since 2015, the entire pancreatic parenchyma was sampled. A median of 31 (range 11–81) formalin-fixed paraffin-embedded (FFPE) blocks were taken for each case.

All available slides were retrieved from the archive and reviewed by two expert pancreatic pathologists (M.S.L., C.D.) blinded to clinical information. FFPE blocks routinely sectioned at 4–5 μm were stained with hematoxylin and eosin (H&E). Slides were evaluated and assigned a score for each of the morphological parameters described below. Discordant scores were reviewed together under a multi-head microscope, and a final consensus between the two pathologists was reached for each case. For uniformity, all cases were reclassified according to the World Health Organization (WHO) Classification of Tumours—Digestive System Tumours, 2019^18^ and restaged according to the eighth edition (2017) of the Union for International Cancer Control (UICC) TNM classification.^19^ The presence of tumor at or within 1 mm of resection margin was assigned as a positive margin (R1)^20^ for all pancreatic margins. The lymph node (LN) ratio was calculated as the ratio of positive LN divided by the total number of sampled LN.

A comprehensive histological assessment was performed evaluating 20 morphological parameters (Table S2). Briefly, dispersion described the residual adenocarcinoma cell distribution in the tumor bed (0: single mass without dispersion; 1: sparse foci in adjacent samples; 2: sparse foci in distant samples) and regressive change described the cytological alterations of carcinoma cells (0: absent; 1: focally present; 2: diffusely present), and neoplastic necrosis was similarly scored. The presence of precursor lesions such as pancreatic intraepithelial neoplasia (PanIN) and intraductal papillary mucinous neoplasm (IPMN)^18^ was assessed and graded as low grade versus high grade.^17,21^ Perineural, lymphovascular, and duodenal invasion by neoplastic cells were all evaluated and scored as dichotomous variables; 0 where absent and 1 when present at any level.

The tumor stroma was evaluated for the presence of keloid-like reaction, hyaline stroma, acellular mucin accumulation, dystrophic calcifications, and cellular stroma. The relationship between stroma and cellular neoplasia was scored according to the prevailing component within the tumor bed (score 1/stroma poor: neoplasia ≥ stroma; score 2/stroma rich: stroma > neoplasia). The presence of vascular wall alterations such as subintimal thickening, undulation of the inner elastic lamina, nonneoplastic thrombosis of the lumen, and changes of the muscular wall were evaluated. The presence of inflammatory cells was scored per cell type: granulocytes, macrophages, and lymphocytes; the latter, when forming architecturally distinct aggregates, were considered as tertiary lymphoid structures (TLS). An assessment of treatment response was also performed using the following published methods: Evans,^10^ College of American Pathologists (CAP),^22^ and M.D. Anderson^23^ tumor regression grading (TRG) systems.

### Statistical Analysis

Disease-free survival (DFS) was defined as the time from treatment start to disease recurrence, death, or last follow-up for censored patient. Overall survival (OS) was defined as the time from treatment start to death or last follow-up for censored patients. Differences between the groups were evaluated using Fisher’s exact test. Interobserver agreement was assessed using Cohen’s kappa coefficient. The association between patient survival and morphological variables was investigated using the Cox proportional-hazards model on univariate analysis (COX-U).

To pre-select the clinical variables best associated with OS and DFS, a machine learning bootstrap-based method, built in the Matlab (v2020b) environment, was used.^24^ In short, the original sample was bootstrapped 1000 times and a COX-U was run for each sample bootstrapped and for each endpoint. The most significant variables occurring in each sample were ranked according to the frequency of their selection among the significantly predictive variables. Two models were developed for OS and DFS.

For each model, the most frequent variables at the top of bootstrap ranking procedure (variables with *p*-value < 0.05 in more than 500 cases of the 1000 bootstrapped samples) were included in a Cox proportional-hazards model on backward multivariate analysis (COX-M) for the prediction of OS and DFS. A *p*-value < 0.20 and a backward selection was set to retain variables in the model.

A prognostic index after neoadjuvant chemotherapy (PINC) was derived for each patient as the risk associated with the selected parameters according to the formula of the Cox regression:

1 $${\text{PINC}} = B0 + \mathop \sum \limits_{1}^{n} Bn*Xn$$

where Bn are the coefficients of COX-M and Xn is the variables selected.

To represent the ability of the PINC in stratifying patients according to their OS and DFS, a cut-off value was derived as the best criterion according to the maximum value of the Youden index of the receiver operating characteristic (ROC) curve, having considered the OS and DFS as independent variables. PINC was then dichotomized as greater and smaller than the cut-off value, and finally the separation of the survival curves between the two groups was tested with the Kaplan–Meier test.

The performance of the models was quantified in terms of the area under the curve (AUC) of ROC curves, based on best cut-off according to the maximum value of the Youden index. Statistical analysis was performed in R (R Core Team, 2019) or MedCalc (v 20.008).

## Results

Between 2005 and 2016, 69 patients with resectable or borderline resectable PDAC received preoperative NACT (neoadjuvant group), while 36 patients with resectable PDAC underwent upfront resection and received postoperative adjuvant therapy (upfront-surgery group). More specifically, at diagnosis, patients with resectable PDAC were randomized to preoperative and postoperative PEXG (cisplatin, epirubicin, capecitabine, gemcitabine) (subgroup A), or to upfront surgery followed by adjuvant PEXG (subgroup B1) or gemcitabine (GEM) (subgroup B2).^4^ Patients with borderline resectable disease, based on historical period, received pre- and postoperative chemotherapy with either PEXG or PDXG (cisplatin, docetaxel, capecitabine, gemcitabine)^15^ (subgroup C) or pre- and postoperative chemotherapy with either PAXG (cisplatin, nab-paclitaxel, capecitabine, gemcitabine)^16,17^ or AG (nab-paclitaxel, gemcitabine) (subgroup D).^17^ Age and sex of patients were equal between the upfront-surgery and neoadjuvant groups, albeit males were overrepresented in subgroup A (Table 1). The distribution of morphological parameters evaluated was significantly different between the upfront-surgery (Groups B1 and B2) and neoadjuvant groups (Groups A, C, and D) (Table 2). Similar results were obtained by analyzing the subset of resectable PDAC patients alone, demonstrating the effect of NACT on tumor morphology (Table S3). The interobserver agreement of the morphological evaluation was substantial [kappa value: 0.72 (95% CI 0.56–0.89)].

**Table 1:** Baseline characteristics

Therapy	Upfront-surgery group (*N* = 36)		Neoadjuvant group (*N* = 69)		
	Adjuvant PEXG Group B1 (*N* = 20)	Adjuvant GEM Group B2 (*N* = 16)	Neoadjuvant PEXG Group A (*N* = 18)	Primary PEXG/PDGX Group C (*N* = 32)	Primary PAXG Group D (*N* = 19)
Sex					
Male	10 (50%)	9 (57%)	15 (84%)	17 (53%)	9 (47%)
Female	10 (50%)	7 (43%)	3 (16%)	15 (47%)	10 (53%)
Age, years	69 (50–75)	67 (37–74)	66 (46–76)	65 (41–74)	69 (51–76)
Tumor location					
Head	16 (80%)	14 (87%)	15 (83%)	25 (78%)	14 (74%)
Body or tail	3 (15%)	1 (6.5%)	3 (17%)	5 (16%)	5 (26%)
Head and body	1 (5%)	1 (6.5%)	0	1 (3%)	0
Head and tail	0	0	0	1 (3%)	0
Tumor					
pT1a	0	0	1 (6%)	1 (3%)	0
pT1b	0	0	2 (11%)	3 (10%)	1 (5%)
pT1c	10 (50%)	2 (12%)	8 (44%)	8 (25%)	7 (37%)
pT2	6 (30%)	12 (76%)	6 (33%)	17 (53%)	8 (42%)
pT3	4 (20%)	2 (12%)	1 (6%)	1 (3%)	1 (5%)
NA	0	0	0	2 (6%)	2 (11%)
Grade					
G1	0	1 (6%)	1 (5%)	2 (6%)	0
G2	8 (40%)	5 (31%)	12 (67%)	15 (47%)	15 (79%)
G3	12 (60%)	10 (63%)	5 (28%)	12 (37%)	2 (11%)
NA	0	0	0	3 (10%)	2 (11%)
Nodes					
N0	5 (25%)	4 (25%)	10 (55%)	13 (41%)	11 (58%)
N1	8 (40%)	4 (25%)	3 (17%)	12 (37%)	7 (37%)
N2	7 (35%)	8 (50%)	5 (28%)	6 (19%)	1 (5%)
NA	0	0	0	1 (3%)	0
Resection margin					
R0	9 (45%)	2 (12%)	12 (67%)	6 (19%)	6 (32%)
R1	11 (55%)	14 (88%)	6 (33%)	26 (81%)	13 (68%)

Sex, age, and tumor location are reported at the time of NACT or surgery. Tumor factor, grade, nodal involvement, and resection margin are evaluated on the histology. *PEXG* cisplatin, epirubicin, capecitabine, gemcitabine, *GEM* gemcitabine, *PDGX* cisplatin, docetaxel, capecitabine, gemcitabine, *PAXG* cisplatin, nab-paclitaxel, capecitabine, gemcitabine

**Table 2:** Morphological features of histological specimens of patients of either neoadjuvant or surgical group

Characteristic	Neoadjuvant	Upfront surgery	*p*-Value^a^
	*N* (%)	*N* (%)	
*Grade^*			
G2	42 (68.9)	13 (37.1)	
G3	19 (31.1)	22 (62.9)	**0.003**
*Nodes*			
pN0	34 (50)	9 (25)	
pN1	22 (32.4)	13 (36.1)	
pN2	12 (17.6)	14 (38.9)	**0.019**
*Dispersion*			
Absent	24 (34.8)	31 (86.1)	
Present	45 (65.2)	5 (13.9)	**< 0.0001**
*Stroma/Neoplasia ratio*			
Neoplasia ≥ stroma	20 (29.4)	30 (83.3)	
Stroma > neoplasia	48 (70.6)	6 (16.7)	**< 0.0001**
*Vascular invasion*			
Absent	39 (56.5)	7 (19.4)	
Present	30 (43.5)	29 (80.6)	**0.0004**
*Granulocytes*			
Absent	48 (69.6)	11 (30.6)	
Present	21 (30.4)	25 (69.4)	**0.0002**
*Hyaline fibrosis*			
Absent	17 (24.6)	25 (69.4)	
Present	52 (75.4)	11 (30.6)	**< 0.0001**
*Necrosis*			
Absent	55 (79.7)	21 (58.3)	
Present	14 (20.3)	15 (41.7)	**0.023**
*Vascular wall alterations*			
Absent	2 (2.9)	7 (19.4)	
Present	67 (97.1)	29 (80.6)	**0.0072**
*Stromal calcification*			
Absent	57 (82.6)	36 (100)	
Present	12 (17.4)	0 (0)	**0.007**
*Regressive changes*			
Absent	25 (36.2)	33 (92)	
Focal	21 (30.4)	3 (8)	
Diffuse	23 (33.4)	0 (0)	**< 0.0001**
*Cellular stroma*			
Absent	51 (73.9)	20 (55.6)	
Present	18 (26.1)	16 (44.4)	0.078
*PanIN/IPMN*			
Absent	18 (26.5)	4 (11.1)	
Present	50 (73.5)	32 (88.9)	0.081
TLS			
Absent	53 (76.8)	32 (88.9)	
Present	16 (23.2)	4 (11.1)	0.19
*Macrophages*			
Absent	41 (59.4)	26 (72.2)	
Present	28 (40.6)	10 (27.8)	0.2
*Perineural invasion*			
Absent	20 (29)	6 (16.7)	
Present	49 (71)	30 (83.3)	0.23
*Lymphocytes*			
Absent	23 (33.3)	16 (44.4)	
Present	46 (66.7)	20 (55.6)	0.29
Duodenal invasion*			
Absent	28 (51)	13 (40)	
Present	27 (49)	20 (60)	0.38
*Mucin*			
Absent	59 (85.5)	32 (88.9)	
Present	10 (14.5)	4 (11.1)	0.77
*Resection margin*			
R0	24 (34.8)	11 (30.6)	
R1	45 (65.2)	25 (69.4)	0.82
*Keloid stromal reaction*			
Absent	41 (59.4)	22 (61.1)	
Present	28 (40.6)	14 (38.9)	> 0.9

*PanIN* pancreatic intraepithelial neoplasia, *IPMN* intraductal papillary mucinous neoplasm, *TLS* tertiary lymphoid structure

^a^Fisher’s exact test, *p* < 0.05. ^G1 were excluded from the analysis

^*^Not evaluated for distal pancreatectomy specimens

For the neoadjuvant group, the univariate analysis indicated LN involvement (HR = 53.3; *p* < 0.0001), necrosis (HR = 3.31; *p* < 0.0001), presence of perineural invasion (HR = 2.81; *p* = 0.006), high tumor grade (HR = 2.09; *p* = 0.025), duodenal invasion (HR = 2.05; *p* = 0.032), and positive resection margins (HR = 1.99; *p* = 0.04) as predictors of shorter OS. Conversely, high stroma-to-neoplasia ratio (HR = 0.38; *p* = 0.002), presence of dispersion (HR = 0.44; *p* = 0.006), diffuse regressive changes (HR = 0.39; *p* = 0.013), and presence of mucin (HR = 0.35; *p* = 0.047) were positive prognostic factors. Similar results were obtained for DFS, with the addition of vascular invasion (HR = 1.75; *p* = 0.046) as a negative prognostic factor (Table 3).

**Table 3:** Univariate Cox regression analysis

Characteristic	Disease-free survival				Overall survival			
	*N*	HR	95% CI	*p*-Value	*N*	HR	95% CI	*p*-Value
Resection margin	68				69			
R0								
R1		1.69	0.92, 3.1	0.088		1.99	1.03, 3.85	**0.040**
Duodenal invasion*	55				55			
Absent								
Present		2.12	1.12, 4.01	**0.021**		2.05	1.06, 3.94	**0.032**
Grade^	60				61			
G2								
G3		1.66	0.9, 3.07	0.1		2.09	1.10, 3.97	**0.025**
Perineural invasion	68				69			
Absent								
Present		2.23	1.16, 4.3	**0.016**		2.81	1.35, 5.86	**0.006**
Necrosis	68				69			
Absent								
Present		2.4	1.25, 4.61	**0.008**		3.31	1.73, 6.37	**< 0.001**
Nodes	67				68			
pN0								
pN1		2.04	1.07, 3.88	**0.029**		2.57	1.30, 5.07	**0.006**
pN2		3.08	1.47, 6.43	**0.003**		2.51	1.18, 5.33	**0.017**
LN ratio	67	17.9	3.26, 97.9	**<** **0.001**	68	53.3	7.60, 374	**< 0.001**
Mucin	69				69			
Absent								
Present		0.37	0.15, 0.94	**0.036**		0.35	0.13, 0.99	**0.047**
Regressive changes	69				69			
Absent								
Focal		0.64	0.33, 1.23	0.2		0.71	0.37, 1.37	0.3
Diffuse		0.48	0.15, 0.94	**0.033**		0.39	0.18, 0.82	**0.013**
Dispersion	68				69			
Absent								
Present		0.54	0.31, 0.95	**0.031**		0.44	0.24, 0.78	**0.006**
Stroma/Neoplasia Ratio	68				68			
Neoplasia ≥ Stroma								
Stroma > Neoplasia		0.5	0.28, 0.9	**0.021**		0.38	0.21, 0.70	**0.002**
Macrophages	68				69			
Absent								
Present		1.23	0.71, 2.14	0.5		1.39	0.77, 2.51	0.3
Vascular invasion	68				69			
Absent								
Present		1.75	1.01, 3.02	**0.046**		1.65	0.93, 2.94	0.086
Vascular wall alterations	68				69			
Absent								
Present		0.42	0.1, 1.77	0.2		0.28	0.07, 1.18	0.083
PanIN/IPMN	67				68			
Absent								
Present		0.76	0.41, 1.4	0.4		0.68	0.35, 1.29	0.2
Keloid stromal reaction	68				69			
Absent								
Present		0.9	0.51, 1.56	0.7		0.83	0.47, 1.49	0.5
Hyaline fibrosis	68				69			
Absent								
Present		0.71	0.38, 1.34	0.3		0.64	0.33, 1.22	0.2
Cellular stroma	68				69			
Absent								
Present		1.32	0.72, 2.44	0.4		1.47	0.76, 2.86	0.3
TLS	68				69			
Absent								
Present		1.8	0.97, 3.34	0.063		1.84	0.92, 3.67	0.084
Lymphocytes	68				69			
Absent								
Present		0.95	0.54, 1.96	0.9		1.01	0.55, 1.83	> 0.9
Granulocytes	68				69			
Absent								
Present		1.63	0.92, 2.9	0.093		1.52	0.84, 2.76	0.2
Stromal calcification	69				69			
Absent								
Present		0.90	0.44, 1.85	0.8		0.97	0.45, 2.09	> 0.9

*HR* hazard ratio, *CI* confidence interval, *LN* ratio lymph nodes ratio, *PanIN* pancreatic intraepithelial neoplasia, *IPMN* intraductal papillary mucinous neoplasm, *TLS* tertiary lymphoid structure

^G1 were excluded from the analysis. *Not evaluated for distal pancreatectomy specimens

**Fig. 1:**
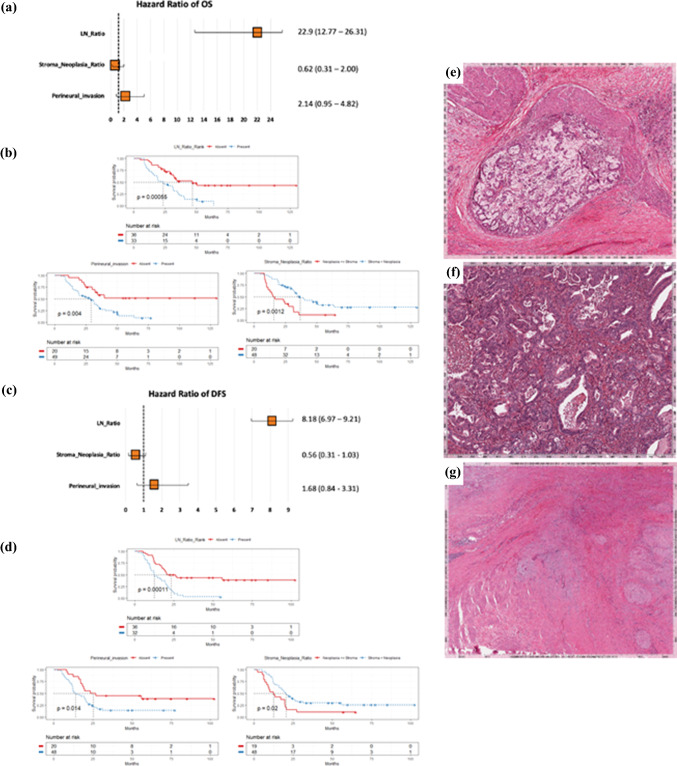
Multivariate Cox proportional hazard model of OS and DFS of patients belonging to neoadjuvant group. (**A**) Forest plot of multivariate analysis based on OS. (**B**) Kaplan–Meier curves of the significant predictive categorical variables based on OS. (**C**) Forest plot of multivariate analysis based on DFS. (**D**) Kaplan–Meier curves of the significant predictive categorical variables based on DFS. (**E**) Representative image of peri- and endoneural invasion. (**F**) Representative image of stroma-poor tumor. (**G**) Representative image of stroma-rich tumor

On multivariate analysis for patients that received NACT, the predictors of shorter OS and DFS were perineural invasion and LN ratio, while high stroma to neoplasia ratio predicted longer OS and DFS (Fig. 1). According to the results of COX-M for OS and DFS, PINCs were computed as follows:

$$ \begin{aligned} {\text{PINC\_OS}} & = 3.1318 \times {\text{LN\_Ratio}} + 0.7647 \times {\text{Perineural\_invasion}} - 0.4788 \times {\text{Stroma\_Neoplasia\_Ratio}} \\ {\text{PINC\_DFS}} & = 2.1019 \times {\text{LN\_Ratio}} + 0.5173 \times {\text{Perineural\_invasion}} - 0.5836 \times {\text{Stroma\_Neoplasia\_Ratio}} \\ \end{aligned} $$

**Fig. 2:**
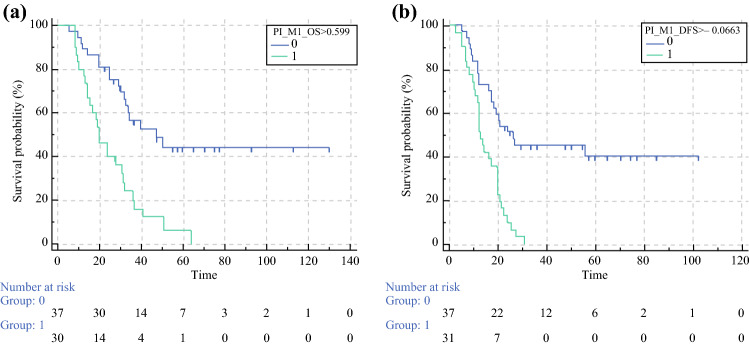
Kaplan–Meier curves based on the prognostic index after neoadjuvant chemotherapy (PINC). (**A**) Based on overall survival (log-rank test, *p* < 0.0001). (**B**) Based on disease-free survival (log-rank test, *p* < 0.0002). 0 = low risk population, 1 = high risk population. The *x*-axis represents the months of OS (left panel) or DFS (right panel)

**Fig. 3:**
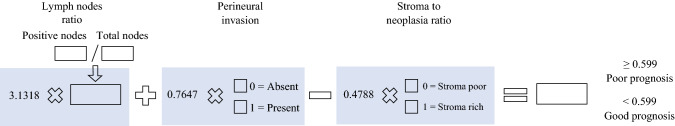
Scheme of the algorithm for determining PINC based on OS

The PINC based on OS and DFS significantly distinguished patients with poor outcome from those with good outcome (*p* < 0.0001 and *p* = 0.0002, respectively). Figure 2 shows the survival curves for OS and DFS after the stratification in the groups with good outcome (PINC_OS < 0.599 and PINC_DFS < −0.066) and the groups with poor outcome (PINCs upper thresholds). The practical algorithm to calculate the PINC is summarized in Fig. 3.

Evans and CAP TRG systems showed a correlation between lower response rates with shorter OS and DFS. However, survival curves showed significant overlaps of the higher regression grades for each system (Figs. S1, S2). Similarly, no correlation was found between M.D. Anderson TRG scores and patient prognosis (Fig. S3).

## Discussion

Growing evidence endorses the use of NACT on patients diagnosed with resectable PDAC compared with upfront surgery, with a possible increase in both DFS and OS.^4^ Indeed, clinical practice is increasingly shifting from a direct surgical approach to a systemic treatment strategy since the priority is to block early metastatic dissemination rather than remove the primary tumor. Consequently, the number of patients undergoing surgery after NACT is expected to increase in the near future. An objective and reproducible histopathologic evaluation of surgical specimens after NACT could play a pivotal role in providing an assessment of therapy-related effects, allowing intertrial comparisons and guiding postoperative treatment choice based on best evidence. Moreover, should a relationship between pathologic response and outcome be demonstrated, the classification would also provide prognostic information and stratify patients in future prospective trials. In addition, a reliable pathologic prognostic index may allow the clustering of patients into different groups of clinical relevance.

Currently, multiple tumor response scoring systems have been proposed,^25^ of which the most widely used in the literature are the Evans,^10^ College of American Pathologists (CAP),^22^ and M.D. Anderson^23^ scoring systems. However, no standardization has yet been achieved in clinical practice. Indeed, the lack of consensus on which score represents best practice, interobserver variability, and reproducibility is a major challenge in the pathologic assessment of response after NACT. Some studies have evaluated the reproducibility of the available grading systems, highlighting a lack of precision, low degree of concordance, and no correlation with prognosis in independent cohorts.^23,26–28^ Furthermore, current response scoring systems do not provide prognostic stratification outside the rare situations of a complete or near complete response.^23,26^ In the current study, none of these TRG systems predicted DFS and OS (Figs. S1, S2, S3).

These scoring systems are based either on the amount of treatment-related fibrosis compared with residual tumor or, alternatively, on the proportion of viable residual tumor compared with the size of tumor bed. In other organs, fibrosis is exploited as a biomarker of response to therapy in different grading systems.^29,30^ However, chemo-naïve PDAC is often associated with an inherent extensive desmoplastic stromal reaction that is almost indistinguishable from therapy-induced fibrosis.^31^ Fibrosis may also be due to associated pancreatitis or be secondary to obstructive ductal changes induced by the tumor mass. In fact, as emerged from a survey conducted among 23 pancreatic pathologists from 4 continents, 87% believed that the amount of fibrosis in comparison to the extent of viable tumor was not a reliable scoring criterion.^13^

In the present work, we performed a comprehensive characterization of the morphological differences presenting in patients who received NACT compared with those who underwent upfront surgery, using 20 different histologic parameters (Table S2). The statistically significant differences in the distribution of numerous morphological parameters between neoadjuvant and upfront surgery patients (Table 2) suggest that chemotherapy caused radical changes in tumor morphology, even though none of these features can be exclusively correlated with the effect of chemotherapy. Therefore, we considered a paradigm shift in the evaluation of patients who received neoadjuvant treatment. Those parameters that were able to predict prognosis after NACT and relatively objective to evaluate were incorporated into a meaningful prognostic index.

Multivariate analysis showed that LN involvement, perineural invasion, and the ratio between residual tumor cells and stroma correlated significantly with patient prognosis. LN involvement was described as a quantitative parameter, calculated as the ratio of positive LN over the total number of sampled LN. Perineural invasion was categorized as a dichotomous variable of absent versus present. The ratio between stroma and residual tumor was based on a qualitative evaluation of the area covered by the two components. In this model, the variable was stratified in two levels: stroma rich tumors, where fibrosis predominated, and stroma poor tumors, where neoplastic cells were equally or more represented than the stromal component. To increase reproducibility, immunostaining of tumor cells and stromal components could be implemented to perform quantitative evaluation of the stroma-to-neoplasia ratio by a simple method of automated immunohistochemistry image analysis. Necrosis was excluded from the grading scheme because of the difficulty in distinguishing treatment-related necrosis from tumor necrosis.

Although the current analysis was carried out on a limited number of patients, relevant morphological features have already been correlated with prognosis, further reinforcing the present findings. For instance, perineural invasion is known to be involved in PDAC dissemination at distant organs, and correlates with poor prognosis.^32^ LN involvement is part of the TNM scoring system and is a well-established prognostic factor. However, LN ratio may be more informative compared with the TNM classification,^33^ even though it may be affected by the total number of LN sampled and assessed. In the present study, 25 nodes (range 5–78) were evaluated on average. Indeed, we confirmed that LN ratio has the strongest association with both DFS and OS in our cohort.

The novelty of the present classification is that perineural invasion, LN ratio, and stroma-to-neoplasia ratio were combined to generate a comprehensive tumor regression scoring system that can be easily translated into clinical practice. Results of our multivariate analysis have been used to generate a prognostic index (PINC), based on the linear combination of Cox coefficients of the selected variables multiplied by the values of the respective variables in the population, thus weighting the influence of each parameter in the formula. The resulting best threshold was applied to stratify the cohort and significantly distinguished patients with good versus poor prognosis.

Despite the promising results, this study has some limitations. First, the number of patients was relatively small over a long time span (2005–2016). Second, different and noncanonical neoadjuvant regimens were used. Because of the limited number of cases, we could not determine the effect of each regimen. Nevertheless, our intent was to create a universal prognostic score that was independent of the chemotherapy regimen. Furthermore, by comparing the distribution of morphological parameters among the different therapeutic subgroups of the neoadjuvant group, we did not identify major differences. To verify the robustness of the PINC, a validation study on a larger cohort of patients will be carried out. In addition, it will be applied in a retrospective series of patients treated with FOLFIRINOX and in the context of a multicenter, prospective clinical trial led by our institution (PACT-21 trial; NCT04793932). The trial will enroll more than 250 patients with resectable or borderline resectable pancreatic cancer that will receive NACT followed by surgery, allowing the evaluation of effective prognostic reliability in a real-life clinical setting and interobserver reproducibility across different centers of the PINC.

In conclusion, the PINC proposed in the current study is mostly quantitative in nature, easy to use, and may represent a reliable tumor regression grading system to predict patient outcomes after NACT followed by surgery for PDAC.

## Supplementary Information

Below is the link to the electronic supplementary material.

Supplementary file1 (DOCX 393 kb)
