# Supplementary material for: Proposal for a New Pathologic Prognostic Index After Neoadjuvant Chemotherapy in Pancreatic Ductal Adenocarcinoma (PINC)
Source: Ann Surg Oncol. 2022 Mar 1;29(6):3492–502. doi: 10.1245/s10434-022-11413-7 (PMC9072515; doi:10.1245/s10434-022-11413-7)
Supplement: Supplementary file 1 — Supplementary file1 (DOCX 393 kb) [file 10434_2022_11413_MOESM1_ESM.docx]

Table S1. Description of the clinical trials enrolling patients included in the study

| Trial name |  | Time of enrolment | Patients | Treatment | Inclusion criteria |
| --- | --- | --- | --- | --- | --- |
| PACT-9  Pancreatic AdenoCarcinoma Trials-9; Clinical- Trials.gov ID: NCT00966706 | open, single institution, randomized phase II trial | July 2005 - September 2008 | pathologically proven stage III–IV pancreatic adenocarcinoma [26], and at least one measurable lesion according to RECIST criteria | PEXG / PDXG | Chemotherapy-naive  18–70 years and KPS>60 or 71–75 years and KPS>80  Adequate bone marrow (absolute neutrophil count (ANC) ≥1,500 cells/mm3; platelet count ≥100,000 cells/mm3; and hemoglobin ≥10 g/dl); kidney (serum creatinine ≤1.5 mg/ dl); and liver function (serum total bilirubin ≤1.5 mg/dl and serum transaminases ≤3 times the upper limit of laboratory normal). |
| ClinicalTrials.gov number, NCT01730222 | single-arm phase 1 study | December 2012 - April 2014 | pathologic diagnosis of unresectable or borderline resectable pancreatic adenocarcinoma, without distant metastases | PAXG regimen | Chemo-naive  18–75 years  KPS>70  Adequate bone marrow (leucocytes ≥3500mm^-3^, absolute neutrophil count ≥1500mm^-3^; platelet count ≥100 000mm^-3^; haemoglobin ≥10 g dl^-1^), liver (total bilirubin ≤2mg dl^-1^;  aspartate aminotransferase and alanine aminotransferase ≤3X upper limit of normal) and kidney function (serum creatinine ≤1.5mg dl^-1^)  Ability to swallow and absorb oral medications |
| PACT-15 trial, ClinicalTrials.gov, number NCT01150630 | randomised, open-label, multicenter, phase 2–3 trial | October 2010 - May 2015 | pathologically  confirmed  pancreatic ductal adenocarcinoma  clinical stage I–II according to the 2010 TNM classification | PEXG / PEFG | Chemo-naive  18–75 years  KPS>60  Resectable disease defined as the absence of invasion of superior mesenteric artery or vein, portal vein, coeliac artery, or hepatic artery;  Adequate bone marrow (white blood cells ≥3500 cells per µL, neutrophils ≥1500 cells per µL, platelets ≥100 000 per µL, and haemoglobin ≥10 g/dL), liver (alanine aminotransferase and aspartate aminotransferase ≤3 upper limit of normal [ULN]), and kidney function (serum creatinine ≤1·5 mg/dL).  The enrolment of patients without a confirmed pathological diagnosis was allowed only when there had been at least one attempt at fine needle aspiration with a negative outcome, and imaging and clinical history were strongly suggestive for the diagnosis of adenocarcinoma |
| NCT01730222 | open-label, single institution, randomised phase II trial | April 2014 -February 2016 | locally advanced or borderline resectable PDAC according to NCCN criteria and as T3 or T4 according to Union for International Cancer Control (UICC)-TNM classification; | PAXG/AG | 18–75 years  KPS was ≥70  Chemo and radio naïve  Adequate bone marrow (GB ≥ 3500/mm3; neutrophils ≥ 1500/ mm3; platelets ≥ 100,000/mm3; haemoglobin ≥10 g/dl), liver (total bilirubin ≤2 mg/dL; SGOT e SGPT ≤3 UNL) and kidney function (serum creatinine ≤1.5 mg/dL). |

Abbreviations: PEXG= cisplatin, epirubicin, capecitabine, gemcitabine; PDGX=cisplatin, docetaxel, capecitabine, gemcitabine; PAXG= cisplatin, nab-paclitaxel, capecitabine, gemcitabine.

Table S2. Description of the morphological parameters assessed

| Parameter | Description | Score |
| --- | --- | --- |
| Grade |  | 0: G2  1: G3  (G1 were excluded because of the small number of samples) |
| Nodes |  | 0: N0  1: N1  2: N2 |
| Resection margin |  | 0: R0  1: R1 |
| Dispersion | Distribution of residual tumor cells in the tumor bed | 0: single mass without dispersion  1: sparse foci in adjacent samples  2: sparse foci in distant samples |
| Stroma to neoplasia ratio | Quantitative relationship between stroma and cellular neoplasia | 0 (Stroma poor): neoplasia ≥ stroma  1 (Stroma rich): stroma > neoplasia |
| Vascular invasion |  | 0: Absent  1: Present |
| Vascular wall alterations | Subintimal thickening, undulation of the inner elastic lamina, non- neoplastic thrombosis of the lumen and changes to the muscular wall | 0: Absent  1: Present |
| Perineural invasion |  | 0: Absent  1: Present |
| Duodenal invasion |  | 0: Absent  1: Present |
| Hyaline fibrosis | Dense fibrotic connective tissue composed of collagen fibers interspersed with few cells | 0: Absent  1: Present |
| Keloid-like reaction | Scar-like fibrotic tissue composed of acellular thick collagen fibers | 0: Absent  1: Present |
| Cellular stroma |  | 0: Absent  1: Present |
| Calcification |  | 0: Absent  1: Present |
| Necrosis |  | 0: Absent  1: Focally present  2: Diffusely present |
| Regressive changes | Cytological alterations of carcinoma cells (nuclei hyperchromasia, cytoplasmic vacuolization) | 0: Absent  1: Focally present  2: Diffusely present |
| Granulocytes |  | 0: Absent  1: Present |
| Lymphocytes |  | 0: Absent  1: Present |
| Macrophages |  | 0: Absent  1: Present |
| TLS |  | 0: Absent  1: Present |
| Mucin |  | 0: Absent  1: Present |
| PanIN/IPMN |  | 0: Absent  1: Low grade  2: High-grade |
| Lymph node ratio | $\frac{N positive lymph nodes}{N lymph nodes sampled}$ | 0 to 1 |

**Table S3**. Morphological features of histological specimens of patients with resectable PDAC of either neoadjuvant or surgical group.

| Characteristic | Neoadjuvant (resectable only) | Upfront-surgery | p- value^1^ |
| --- | --- | --- | --- |
|  | N (%) | N (%) |  |
| Grade^ |  |  |  |
| **G2** | 12 (70.6) | 13 (37.1) |  |
| **G3** | 5 (29.4) | 22 (62.9) | **0.038** |
| Nodes |  |  |  |
| **pN0** | 10 (55.6) | 9 (25) |  |
| **pN1** | 3 (16.7) | 13 (36.1) |  |
| **pN2** | 5 (27.8) | 14 (38.9) | 0.07 |
| Dispersion |  |  |  |
| **Absent** | 4 (22.2) | 31 (86.1) |  |
| **Present** | 14 (77.8) | 5 (13.9) | **<0.0001** |
| Stroma/Neoplasia Ratio |  |  |  |
| **Neoplasia ≥ Stroma** | 5 (27.8) | 30 (83.3) |  |
| **Stroma > Neoplasia** | 13 (72.2) | 6 (16.7) | **<0.0001** |
| Vascular invasion |  |  |  |
| **Absent** | 7 (38.9) | 7 (19.4) |  |
| **Present** | 11 (61.1) | 29 (80.6) | 0.18 |
| Granulocytes |  |  |  |
| **Absent** | 11 (61.1) | 11 (30.6) |  |
| **Present** | 7 (38.9) | 25 (69.4) | **0.042** |
| Hyaline fibrosis |  |  |  |
| **Absent** | 6 (33.3) | 25 (69.4) |  |
| **Present** | 12 (66.7) | 11 (30.6) | **0.018** |
| Necrosis |  |  |  |
| **Absent** | 18 (100) | 21 (58.3) |  |
| **Present** | 0 (0) | 15 (41.7) | **0.0009** |
| Vascular wall alterations |  |  |  |
| **Absent** | 1 (5.6) | 7 (19.4) |  |
| **Present** | 17 (94.4) | 29 (80.6) | 0.24 |
| Stromal calcification |  |  |  |
| **Absent** | 16 (88.9) | 36 (100) |  |
| **Present** | 2 (11.1) | 0 (0) | 0.1 |
| Regressive changes |  |  |  |
| **Absent** | 6 (33.3) | 33 (92) |  |
| **Focal** | 7 (38.9) | 3 (8) |  |
| **Diffuse** | 5 (27.8) | 0 (0) | **<0.0001** |
| Cellular stroma |  |  |  |
| **Absent** | 16 (88.9) | 20 (55.6) |  |
| **Present** | 2 (11.1) | 16 (44.4) | **0.016** |
| PanIN/IPMN |  |  |  |
| **Absent** | 3 (16.7) | 4 (11.1) |  |
| **Present** | 15 (83.3) | 32 (88.9) | 0.67 |
| TLS |  |  |  |
| **Absent** | 17 (94.4) | 32 (88.9) |  |
| **Present** | 1 (5.6) | 4 (11.1) | 0.65 |
| Macrophages |  |  |  |
| **Absent** | 12 (72.2) | 26 (72.2) |  |
| **Present** | 6 (27.8) | 10 (27.8) | 0.75 |
| Perineural invasion |  |  |  |
| **Absent** | 7 (38.9) | 6 (16.7) |  |
| **Present** | 11 (61.1) | 30 (83.3) | 0.096 |
| Lymphocytes |  |  |  |
| **Absent** | 7 (38.9) | 16 (44.4) |  |
| **Present** | 11 (61.1) | 20 (55.6) | 0.29 |
| Duodenal invasion* |  |  |  |
| **Absent** | 9 (60) | 13 (40) |  |
| **Present** | 6 (40) | 20 (60) | 0.22 |
| Mucin |  |  |  |
| **Absent** | 14 (77.8) | 32 (88.9) |  |
| **Present** | 4 (22.2) | 4 (11.1) | 0.41 |
| Resection margin |  |  |  |
| **R0** | 12 (66.7) | 11 (30.6) |  |
| **R1** | 6 (33.3) | 25 (69.4) | **0.018** |
| Keloid stromal reaction |  |  |  |
| **Absent** | 4 (22.2) | 22 (61.1) |  |
| **Present** | 14 (77.8) | 14 (38.9) | **0.009** |

^1^, Fisher’s exact test, *P*<0.05. ^, G1 were excluded from the analysis. *, not evaluated for distal pancreatectomy specimens. Abbreviations: PanIN=pancreatic intraepithelial neoplasia; IPMN=intraductal papillary mucinous neoplasm; TLS=tertiary lymphoid structure.


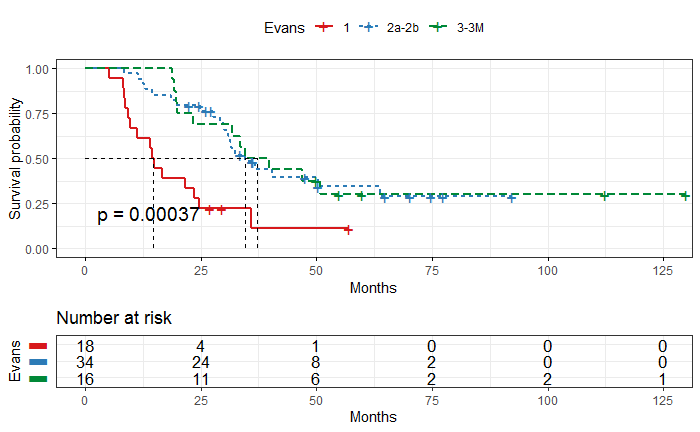

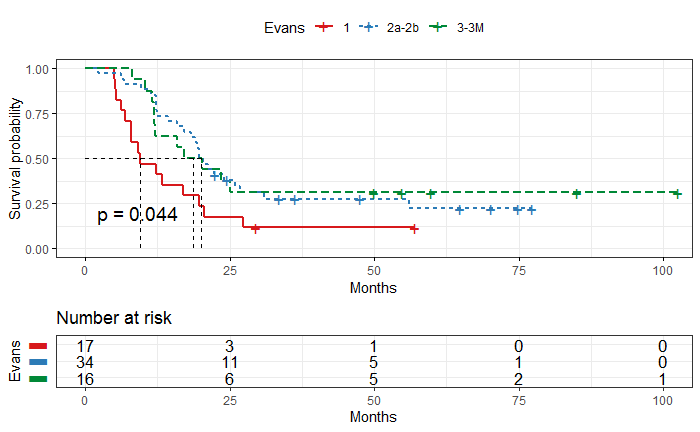


B

A

Figure S1. A. OS prediction after resection stratified by Evans’ score. B. DFS prediction after resection stratified by Evans’ score.


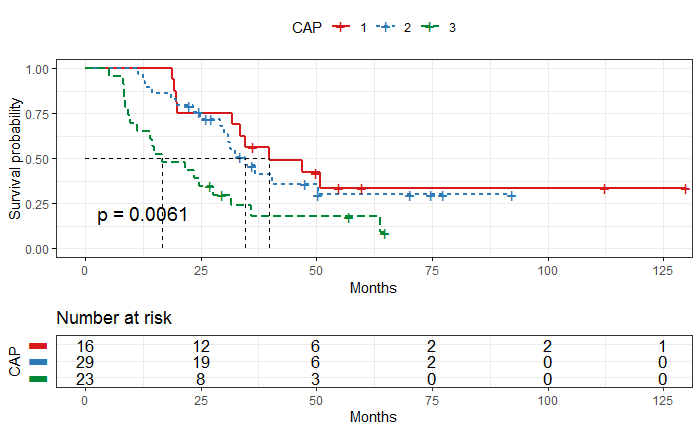

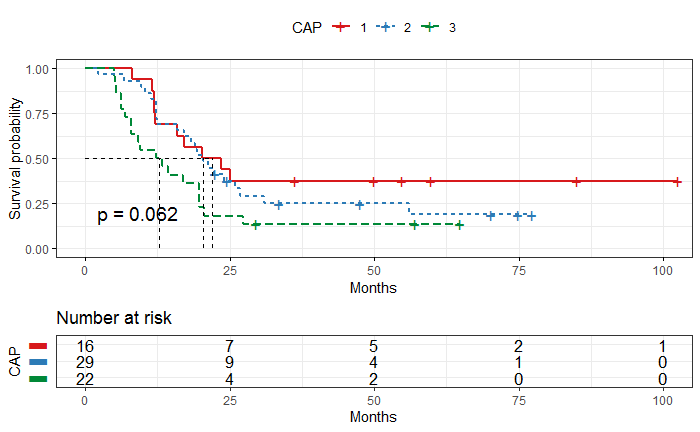


B

A

Figure S2. A. OS prediction after resection stratified by CAP score. B. DFS prediction after resection stratified by CAP score. Abbreviations: CAP=College of American Pathologists


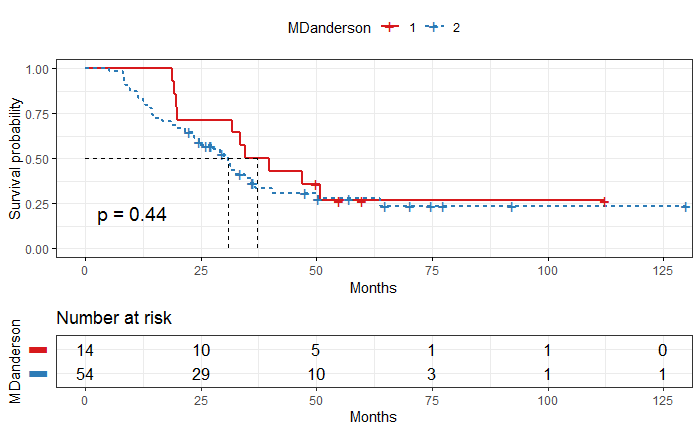

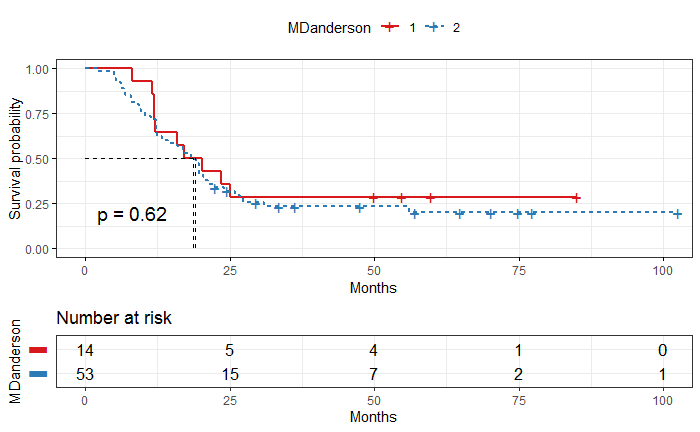


B

A

Figure S3. A. OS prediction after resection stratified by MD Anderson score. B. DFS prediction after resection stratified by MD Anderson score.
